# Indar Kumar Dhawan: A Versatile Master Surgeon and a Gentleman

**DOI:** 10.1055/s-0045-1809705

**Published:** 2025-07-01

**Authors:** Rakesh Kumar Khazanchi

**Affiliations:** 1 Department of Plastic, Aesthetic and Reconstructive Surgery, Medanta the Medicity, Gurugram, Haryana, India

I first met Professor Indar Kumar Dhawan in March 1984, about a year after finishing my superspeciality training in plastic surgery while struggling to find a job. I was nervous and overwhelmed by the occasion. Little did I know that the person I was meeting that day would play a major role in shaping the rest of my career. The meeting lasted just approximately 5 minutes during which I struggled to hear his softly spoken words that the All India Institute of Medical Sciences (AIIMS) did not have a plastic surgery department, but I could consider working as a Pool Officer. I later found out that it was a temporary job funded by the Council of Scientific and Industrial Research (CSIR) to give time to unemployed specialists like me to find a proper job while making two ends meet.

**Fig. 1:**
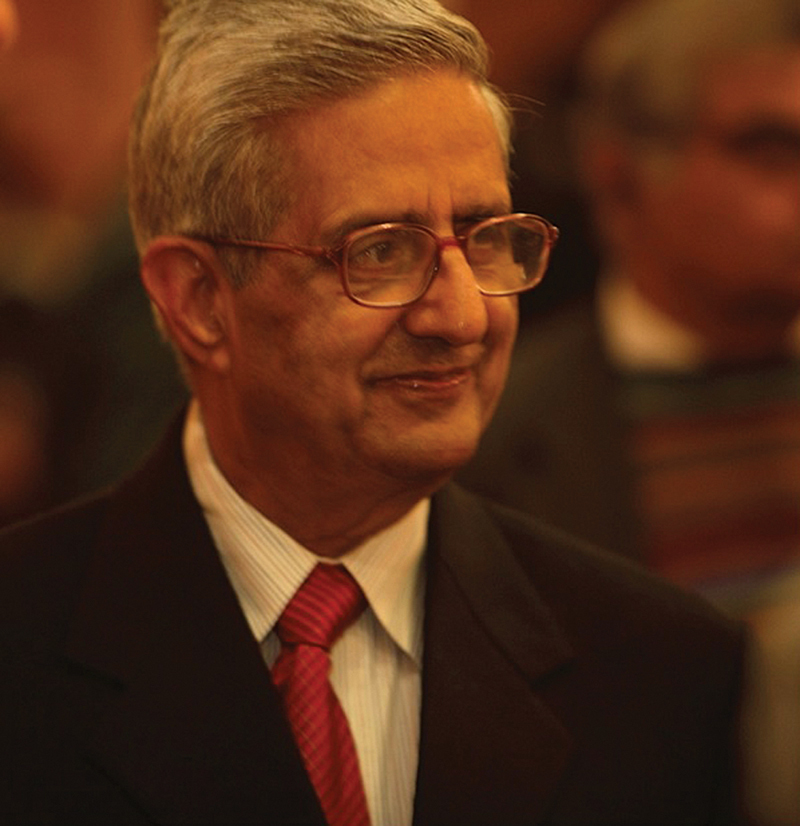
Professor Indar Kumar Dhawan.

Professor Indar Kumar Dhawan ( Fig. 1 ), popularly known as IKD, lived through an era when surgery as a specialty was evolving into superspecialities. It is amazing how he could seamlessly execute and blend his surgical skills across multiple superspecialities, that is, plastic and reconstructive surgery, craniofaciomaxillary surgery, surgical oncology, oncoreconstructive surgery, and renal transplantation in addition to general surgery. IKD was truly a master of all trades. Moreover, he had a humility that was just as unusual. While skillful surgeons of that era were generally known to be egoistic and prone to throwing tantrums in operating rooms, IKD was an epitome of calmness and a thorough gentleman. His exceptional surgical skills and cool demeanor were qualities that defined him and endeared him to his teachers, colleagues, peers, friends, students, staff, and patients. Or really anyone who crossed paths with him.

**Fig. 2:**
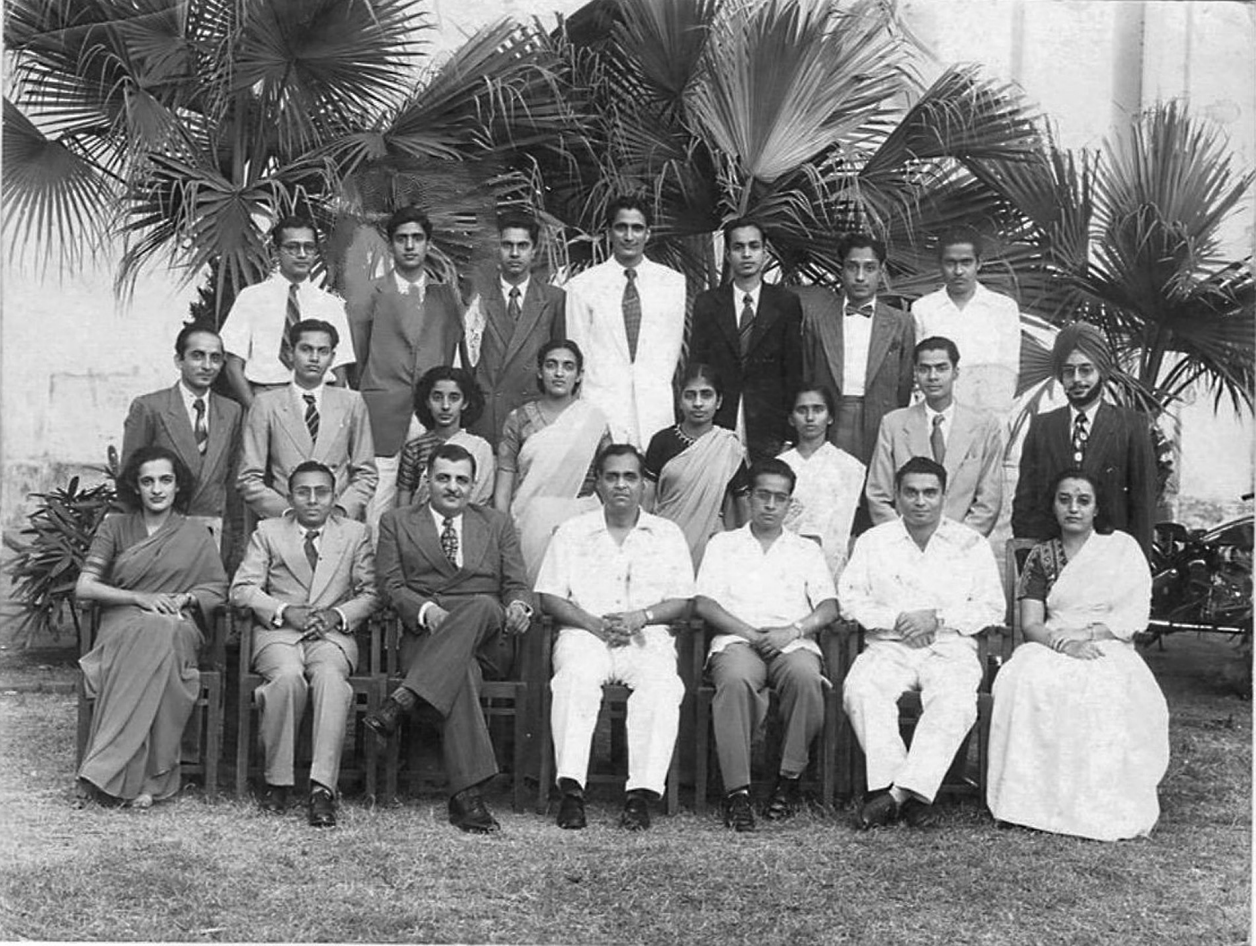
Indar Kumar Dhawan (Upper row, 2nd from left) at Grant Medical College – Mumbai.

IKD was borne in Shimla in 1930 to a Punjabi family with roots in present-day Pakistan. He was nicknamed “Bhola,” a name which proved to be prophetic of his nature during his adult life. When he was 10, the family moved to Delhi where he attended St. Columba's School. For his intermediate, IKD attended Forman Christian College, Lahore. Partition forced his departure to Mumbai (then Bombay) for his MBBS and MS (Surgery) from the famous Grant Medical College ( Fig. 2 ). IKD's academic career was studded with numerous awards and gold medals. While moving overseas to a high salary and large research grants was tempting to many at that time in India, IKD, who was 17, when India became independent, wanted to assist his new country and Nehru's vision of creating a modern and successful country with world-class institutions.

IKD was also a keen sportsman and played football for his college. No matter how busy his life was, he followed every cricket match passionately. In 1958, he joined Irwin (now Lok Nayak Jai Prakash) Hospital, Delhi, as a Surgical Registrar. This is where he honed his surgical skills under two legendary general surgeons—Dr. K. C. Mahajan and Dr. S. K. Sen, and also where he met his life partner Dr. Sushila Sarin, who had recently moved to Delhi after completing her MD in England. He was taken by this pants-wearing, English-language music listening anesthesiologist, and they got married in 1961.

That same year, he joined AIIMS, Delhi, as an Assistant Professor of Surgery. His hard work, exemplary surgical, teaching, and research skills resulted in him being promoted to Associate Professor and Head of Surgery Unit II in 1970. In 1965, he went to the border during the India-Pakistan war and headed a surgical unit to take care of civilian casualties—an appointment he kept from his mother so she wouldn't worry. In 1979, he was promoted to the position of Professor of Surgery and was designated the Head of the Department of Surgical Disciplines in 1982, a position he occupied till he superannuated in 1988. Following his superannuation from the AIIMS, he joined Mafraq Hospital in Abu Dhabi, United Arab Emirates, and was the Chairman of Department of Surgery at that hospital till 1996 when he returned to India. He continued working at Batra Hospital and Sitaram Bhartia Institute in Delhi. His love for teaching continued and he started Diplomate of National Board (DNB) (Surgery) program at Batra Hospital. He worked 6 days a week well into his 90s, till the coronavirus disease lockdown forced him to stay home. But even that didn't stop him. A voracious reader and writer, at the time of his passing, IKD was writing a book on Mahatma Gandhi, whom he greatly admired. He remained active and dedicated to teaching and research till his health started failing and passed away in 2021.

**Fig. 3:**
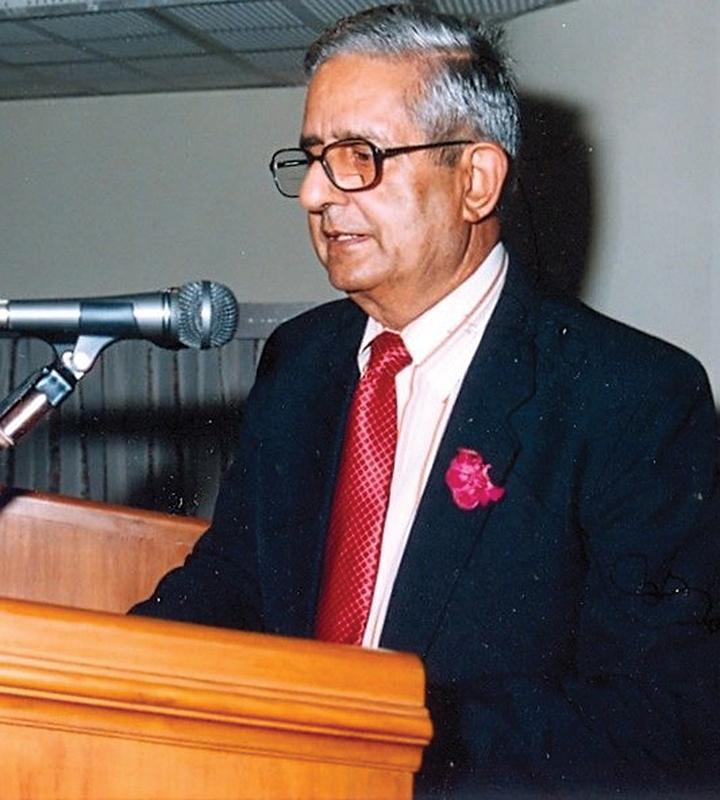
Indar Kumar Dhawan (IKD) delivering Sandoz Oration.

During his brilliant career, he earned many laurels, both in India and internationally. He published numerous papers and authored the “Textbook of Surgery” for undergraduate medical students. He was also honored with positions of President of Association of Plastic Surgeons of India (APSI) (1978), President Delhi Chapter of Association of Surgeons of India, and President of Indian Society of Oncology. His other honors included Sandoz Oration for Cancer Research by Indian Council of Medical Research ( Fig. 3 ), and appointment as “Surgeon to the President of India.” In 2015, he was awarded a lifetime achievement award for Service to Humanity by Prime Minister Modi.

**Fig. 4:**
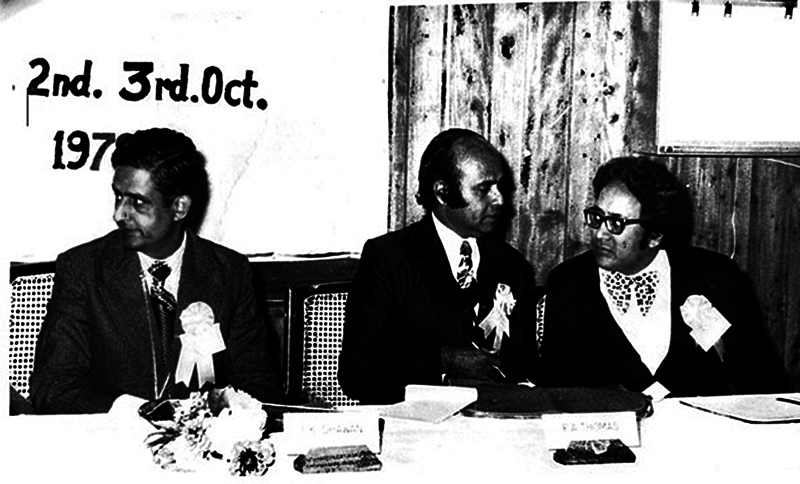
Indar Kumar Dhawan (IKD) at 13th National Conference of Association of Plastic Surgeons of India, 1978.

While IKD was primarily a general surgeon and had no formal degree in plastic surgery, his interest in plastic surgery developed early in his career. In his later years he proudly recounted to this author about his attending the first summer conference of APSI held in Nagpur in 1964 where all of about twenty or so delegates were housed in the newly constructed plastic surgery ward. In 1966, he was awarded Senior Commonwealth Fellowship to train in renal transplantation in the United Kingdom. In the later part of this fellowship he visited plastic surgery centers in the United Kingdom, Sweden, and erstwhile Yugoslavia. In Sweden, he acquired plastic surgery skills from the legendary Dr. Tord Skoog. Back in India, while on one hand he established the Renal Transplant Unit at AIIMS, he also persisted with his interest in plastic surgery. He developed different types of flaps in head and neck reconstruction, 1 2 which are also acknowledged in the “Grabb's Encyclopedia of Flaps.” 3 4 5 In addition, he did a lot of work in cleft 6 and craniofacial surgery 7 in collaboration with dental, ear, nose, and throat, and neurosurgeons. He was one of the first few surgeons who initiated pediatric craniofacial surgery in North India and did many complex surgeries for congenital craniofacial anomalies. 8 In 1978, he hosted the 13th National Conference of Association of Plastic Surgeons of India ( Fig. 4 ). His intense love for plastic surgery is also confirmed by the fact that he was a regular speaker at international plastic surgery conferences in Rome (1967), Melbourne (1971), Paris (1975), Montreal (1983), and New Delhi (1987).

**Fig. 5:**
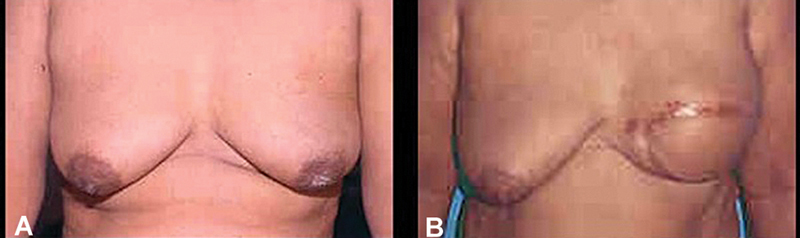
Breast reconstruction for cancer in 1984. ( **A** ) Preop. ( **B** ) Postop.

I joined his unit as a Pool Officer in 1984 with a brief to establish reconstructive microsurgery in the department. It was under his guidance and encouragement that I started to learn microsurgery in the animal laboratory at the AIIMS and followed it with clinical applications from 1985 onwards. In late 1984, I also had the privilege of assisting him in a live video demonstration (probably for the first time in India) of a “latissimus dorsi myocutaneous flap with implant”-based breast reconstruction in a patient with breast cancer, during a conference of Indo-Australian Surgeons at the AIIMS, New Delhi ( Fig. 5 ). While he sowed seeds of plastic surgery at the AIIMS, the author being its first beneficiary, it was much later in 2012, that a department of plastic surgery was established in AIIMS, Delhi.

In addition to being a highly skilled surgeon, he was an accomplished and dedicated teacher, researcher, and administrator, admired equally by his students and peers. Gentle, kind, and encouraging, he was extremely soft spoken and never known to raise his voice. I heard of an incident of his younger days when at the end of an ear reconstruction for microtia, the entire dressing and construct came off during a stormy extubation. He remained unflustered and continued immediately by redoing the reconstruction as if it was the first time. Every evening, after dinner, he went back to the hospital to do nightly rounds and check on his patients, often surprising new residents who didn't expect the head of surgery to make rounds late into the night.

In spite of shouldering numerous responsibilities, which go with the profession and position in the country's premier institute, and dealing with endless stream of VVIPs of the national capital, he never appeared stressed and yet maintained the highest quality of work and ethics. After spending the entire day doing clinical, teaching, and administrative work, he would work in his office till late hours. I once asked for his “Mantra” of staying calm under such extreme circumstances and yet delivering quality care with hard work and dedication? “Strive for perfection but don't be a perfectionist” he replied. In today's world where medical care can often be commercialized and impersonal, he showed us that this was not just a career but a calling. A calling to care for those in need, one that required sacrifice, selflessness, and relentless hard work. All of us who had the good fortune of sharing his journey will always cherish his guidance and legacy.
